# Efficacy of a Mobile Serious Game (SwaziYolo) for Increasing HIV Risk Perception: Randomized Controlled Trial

**DOI:** 10.2196/70333

**Published:** 2025-11-24

**Authors:** Bhekumusa Wellington Lukhele, Mac Delay, Fortunate Shabalala, Mfundi Motsa, Alexander Kay, Christina El Saaidi, Masako Kihara, Masahiro Kihara, Katia J Bruxvoort

**Affiliations:** 1 University of Alabama at Birmingham Birmingham, AL United States; 2 University of Eswatini Mbabane Swaziland; 3 Ministry of Health Mbabane Swaziland; 4 Baylor College of Medicine Houston, TX United States; 5 City of San Antonio Metropolitan Health District San Antonio, TX United States; 6 Kyoto University Kyoto Japan

**Keywords:** HIV prevention, youth, digital health, gamification, mobile health, mHealth, Swaziland, Eswatini

## Abstract

**Background:**

Eswatini has one of the highest HIV prevalence rates worldwide (24.8% among people aged ≥15 years), with unprotected heterosexual transmission accounting for more than 90% of new HIV infections in the country. Low HIV risk perception is known to influence risk behavior. Mobile phone technology is growing rapidly, offering opportunities for technology-driven interventions to improve HIV risk perception and prevention.

**Objective:**

We aimed to design and test a serious game to increase HIV risk perception and intention to engage in protective HIV behaviors among young people in Eswatini.

**Methods:**

Our team developed SwaziYolo, a smartphone-based, interactive, educational story game that places the player in the role of a young adult looking for love in Eswatini’s capital city. We conducted the Serious Games HIV Prevention Trial (SGPrev-Trial), a 4-week, 2-arm, unblinded, 1:1 randomized controlled trial of SwaziYolo among people aged between 18 and 29 years in Eswatini. The primary outcome was HIV risk perception using a 10-item and subset 8-item Perceived Risk of HIV Infection Scale (PRHS). We used modified intention-to-treat and per-protocol difference-in-difference (DID) estimation to compare the change between groups in the mean PHRS scores before and after intervention.

**Results:**

Of the 380 participants in the study, 122 (64.2%) in the control group and 119 (62.6%) in the intervention group completed the follow-up, and 95 (79.8%) played the game. In the modified intention-to-treat analyses, no significant differences between groups were observed for the 8-item PRHS (DID: mean 1.1, SD 0.72; *P*=.13) and the 10-item PRHS (DID: mean 1.3, SD 0.80; *P*=.12) scores. In the per-protocol analyses, HIV risk perception increased significantly among participants who played the game (8-item DID: mean 1.6, SD 0.74; *P*=.04 and 10-item DID: mean 1.8, SD 0.83; *P*=.03). Nearly all (94/95, 98.9%) participants strongly agreed or agreed that they would recommend SwaziYolo to their peers.

**Conclusions:**

SwaziYolo was acceptable and increased HIV risk perception among young people in Eswatini who self-selected to play the game. More research is needed to improve and evaluate the SwaziYolo intervention.

**Trial Registration:**

UMIN Clinical Trial Registry UMIN000021781; https://center6.umin.ac.jp/cgi-open-bin/ctr_e/ctr_view.cgi?recptno=R000025103

## Introduction

The HIV epidemic remains a major public health concern worldwide. In 2023, there were approximately 1.3 million new infections, 3 times more than the estimate for 2025 [1]. The countries with the highest HIV prevalence are in southern Africa, including Eswatini. A 2021 national survey in Eswatini found that the prevalence of HIV among people aged 15 years or older was 24.8% [2]. Unprotected heterosexual transmission accounts for more than 90% of all new infections in Eswatini [3], and multiple concurrent sexual partnerships, low HIV testing, and low HIV risk perception are the key drivers of transmission in the country [3-5]. Antiretroviral treatment aids in prevention. Pre-exposure prophylaxis (PrEP), postexposure prophylaxis, and medical male circumcision have helped reduce HIV incidence. However, there is an urgent need for innovative interventions to prevent HIV infection, particularly among young people [1].

Serious games, commonly referred to as gaming for education, motivation, or behavioral change rather than mere entertainment [6-8], are promising strategies for behavior change interventions. Several studies adopting serious games or gamified interventions have observed effective behavioral change outcomes. For example, a randomized controlled trial (RCT) called PlayForward implemented in the United States found improvements in attitudes toward sexual health and an increase in sexual health knowledge [9]. Other RCTs conducted in Kenya, Tanzania, and South Africa found that serious games were associated with significant improvements in self-efficacy, sexual health knowledge and literacy, sexual risk communication, significant changes in behavioral intention to reduce and avoid HIV risk, and higher cognitive affirmative attitudes [10-13].

Low HIV risk perception is associated with an increased risk of HIV infection [14-16], which supports the need for behavior change interventions to focus on increasing HIV risk perception. A large longitudinal study found that increased HIV risk perception was associated with higher odds of increasing protective behavior, such as condom use [17]. Most behavioral theories include the concept of risk perception, which is believed to be necessary for behavior change [18-20]. Previous studies in Eswatini indicate a low-risk perception, particularly among young adults [21].

Despite the potential of serious games in both high-income and low- or middle-income countries to impact perceptions and behaviors, potentially leading to improvements in health outcomes, few studies have examined serious games for HIV prevention, and none have examined serious games for HIV prevention in Eswatini. To address this gap, we designed and tested a serious game to increase HIV risk perception and intention to engage in protective HIV behaviors among young people in Eswatini.

## Methods

### Study Design and Participants

The Eswatini (formerly Swaziland) Serious Games HIV Prevention Trial (SGPrev-Trial) was a 4-week, 2-arm, unblinded, 1:1 RCT of SwaziYolo, a serious games mobile health intervention that we developed for young people in Eswatini. Details of the intervention mapping steps, game design, game screenshots, theoretical framework adopted, study design, ethical considerations, and HIV risk perception scales are detailed in our published trial protocol [6]. Briefly, the trial was powered to detect a difference in the change in HIV risk perception scores before and after the intervention, between the intervention and control groups at 4 weeks. Assuming a moderate effect size of 0.477 based on the study by Chu et al [22], with an α of .05 (2-tailed) and a β of .20, the required total sample size was 146 per group. To account for an anticipated 30% loss to follow-up [23], we increased the sample size by 30%, resulting in 190 participants per group for a final sample size of 380 participants.

### Intervention Description

**Figure 1:**
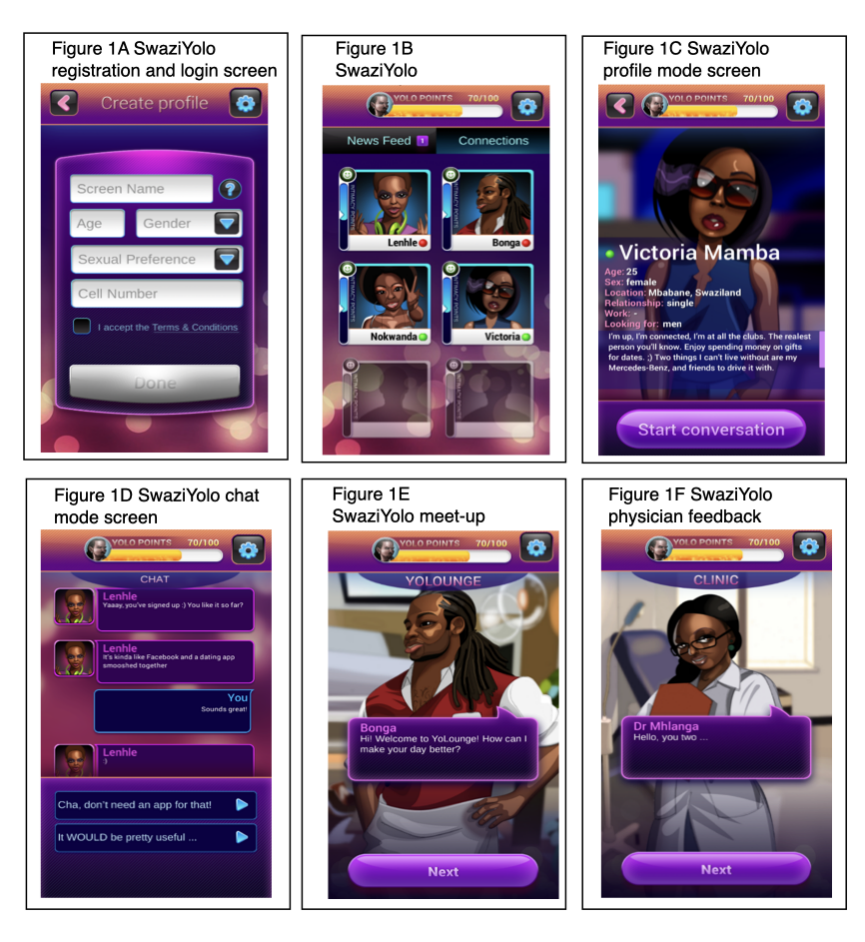
SwaziYolo screenshots.

On the basis of our formative research, SwaziYolo was an interactive, educational story game for mobile phones that placed the player in the role of a young adult looking for love in Eswatini’s capital city. Players were tasked with making important choices regarding relationships and sexual health, as previously described [6]. SwaziYolo incorporates elements of serious games, such as immersion, role-playing, and a scripted, predetermined storyline that varies based on players’ previous selections [6]. The first part of the game was set in an imaginary social network called SwaziYolo, resembling common social media apps. Here, players registered (registration and login screen), viewed profile pictures (potential love interest connections screen) and profiles of potential love interests (profile mode screen), and had web-based chats (chat mode screen) with various characters. The second part of the game took place in various made-up locations around the capital city of Eswatini, such as nightclubs and cafes (meet up at a club screen), where players would go on dates (meetups). In both parts of the game, players were required to make choices to advance conversations or storylines with a friend or love interest (chat mode screen). These choices influenced the opinions and behavior of other characters, as well as the characters’ health and safety. At the end of the game, feedback was provided based on the player’s choices (feedback from a physician at a clinic screen) [6]. Figure 1 shows selected screenshots of SwaziYolo, including the registration and login screen, the potential love interests’ connections screen, the profile mode screen, chat mode screen, meet up screen, and feedback screen.

### Recruitment, Enrollment, and Randomization

Participants were recruited from April 28, 2017, to July 06, 2017. The game was downloadable from the project website and Google Play Store. We also distributed the SwaziYolo app through social media, through popular mobile phone shops, and at universities in major cities around Eswatini [6]. Once the game was downloaded, potential participants were directed to the study information page on our website, and eligibility screening was conducted through a self-administered questionnaire. The eligibility criteria were as follows: aged between 18 and 29 years, owned a smartphone running an Android-based operating system, had WhatsApp, lived in Eswatini at the time of the study, and could adequately grant informed consent. Potential participants who responded yes to all eligibility screening questions (detailed further in the published protocol [6]) were directed to the informed consent page.

We generated 380 5-digit random numbers using Microsoft Excel “=RAND ()” function to serve as one-time passcodes (OTPs). After generating the OTPs, we checked for and removed duplicates. Once participants provided written informed consent, they were each randomized by a computer-generated algorithm that assigned an OTP indicating group assignment. Participants in the intervention group received OTPs that immediately unlocked the SwaziYolo intervention after completing the baseline survey. In contrast, participants in the control group (waitlist group) received OTPs that would only unlock the intervention after completing a follow-up survey after 4 weeks from the trial registration date.

### Data Collection

Participants completed a brief web-based self-administered questionnaire at baseline (before intervention) and then 4 weeks after completion of the baseline survey (after intervention), during which those randomized to the intervention group were able to play the game. The questionnaire included a self-administered, structured, web-based questionnaire created based on a review of the literature and our formative work. For example, questions relating to sociodemographic characteristics were adopted from the 2007 Swaziland Demographic Health Survey, and those related to sexual behaviors and intention to change behaviors were developed from our previous work in Eswatini [21,24].

### Perceived Risk of HIV Infection Scale Outcomes

The primary outcome of interest, HIV risk perception, was measured through the validated Perceived Risk of HIV Infection Scale (PRHS) [20]. We used a 10-item index and an 8-item index (a subset of the 10-item index), as shown in Table 1. Although the 10-item scale has been found to perform as well as the 8-item scale [20], we used both scales to capture additional dimensions of risk perception that may be present in our population. Each Likert-type question was scored (see Table 1 for details) such that the final score was the sum of all 8 or 10 items. We also assessed secondary outcomes, including the number of sexual partners in the last 30 days, condom use in the last 30 days, HIV testing in the last 30 days or intention to test for HIV, intention to know the partner’s HIV status, intention to reduce multiple sexual partnerships, and intention to use a condom in the next sexual encounter. Details of the administered questionnaire are shown in Multimedia Appendix 1.

**Table 1:** The 8-item and 10-item Perceived Risk of HIV Infection Scale [20].

			Item content		Response options
**Items on both the 8-item and 10-item** **Perceived Risk of HIV Infection Scales**					
	Item 1	What is your gut feeling about how likely you are to get infected with HIV?		Extremely unlikely (0), very unlikely (1), somewhat likely (2), very likely (3), or extremely likely (4)	
	Item 2	I worry about getting infected with HIV		None of the time (0), rarely or some of the time (1), a moderate amount of time (2), a lot of the time (3), or all the time (4)	
	Item 3	I am sure I will NOT get HIV infected		Strongly disagree (5), disagree (4), somewhat disagree (3), somewhat agree (2), agree (1), or strongly agree (0)	
	Item 4	I feel vulnerable to HIV infection		Strongly disagree (0), disagree (1), somewhat disagree (2), somewhat agree (3), agree (4), or strongly agree (5)	
	Item 5	There is a chance, no matter how small, that I could get HIV		Strongly disagree (0), disagree (1), somewhat disagree (2), somewhat agree (3), agree (4), or strongly agree (5)	
	Item 6	I think my chances of getting infected with HIV are		Zero (0), almost zero (1), small (2), moderate (3), large (4), or very large (5)	
	Item 7	Picturing myself getting HIV is something I find		Very hard to do (0), hard to do (1), easy to do (2), or very easy to do (3)	
	Item 8	Getting HIV is something I have		Never thought about (0), rarely thought about (1), thought about some of the time (2), or thought about often (3)	
**Items on the 10-item** **Perceived Risk of HIV Infection Scale** **only**					
	Item 9	Getting HIV is something I am		Not concerned about (0), a little concerned about (1), moderately concerned about (3), concerned about a lot (4), or extremely concerned about (5)	
	Item 10	I feel I am unlikely to get infected with HIV		Strongly disagree (5), disagree (4), somewhat disagree (3), somewhat agree (2), agree (1), or strongly agree (0)	

### Statistical Methods

All data analyses were conducted using SAS 9.4. We described the baseline characteristics of individuals in each group and calculated the mean 8-item and 10-item scores and the proportions with each secondary outcome. For the 8-item and 10-item outcomes, we then conducted difference-in-difference (DID) estimation for the change in prescores and postscores comparing the intervention and control groups using mixed effects linear regression. The primary analysis was based on a modified intention-to-treat (mITT) approach due to postrandomization exclusion of participants lost to follow-up, those with missing data, and those who reported a known HIV positive status at baseline and were unlikely to benefit from the intervention. We also conducted per-protocol (PP) analyses with those who played the game in the intervention group, and subgroup analyses using the same DID approach among participants with multiple sexual partners, those without multiple sexual partners, those who reported condomless sex in the past 30 days, and those who did not report condomless sex in the past 30 days.

To compare pre-post differences in secondary outcomes between the intervention and control groups, we used general logistic regression models with the postresponse (yes or no or yes, no, or not sure) as the outcome, the intervention versus control group as the predictor, and the preresponse as a confounder.

### Ethical Considerations

This study was reviewed and approved by the Swaziland Scientific and Ethical Committee (MH/599C/FWA00015267/IRB0009688) and registered at the UMIN Clinical Trial Registry (UMIN000021781). The protocol [6] was written in accordance with the CONSORT-EHEALTH (Consolidated Standards for Reporting Trials of Electronic and Mobile Health Applications and Online Telehealth) checklist [25]. Informed consent was obtained from all participants online before enrollment in the study. Privacy and confidentiality were maintained through the deidentification of data and storage in encrypted servers. Participants’ phone numbers were deleted from the data. A lottery-based incentive with a 1:100 chance of receiving US $20 was given to all participants after baseline data collection. Identification of individual participants was not possible in the manuscript or the Multimedia Appendices.

## Results

### Overview

**Figure 2:**
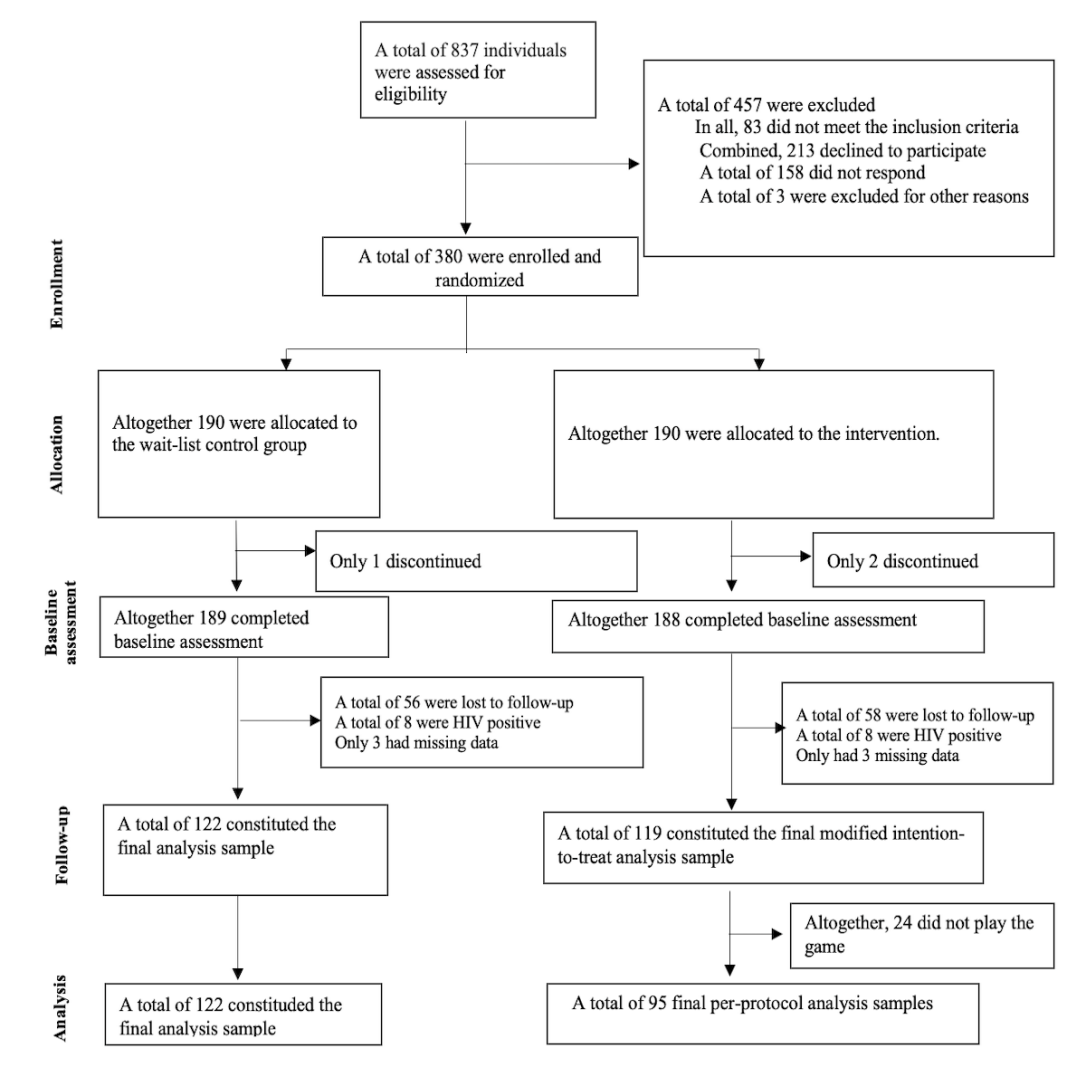
CONSORT (Consolidated Standards of Reporting Trials) diagram for the Serious Games HIV Prevention Trial.

The last participant completed the 4-week follow-up period on August 10, 2017. In total, 837 potential study participants were assessed for eligibility, and 457 (54.5%) declined to participate or did not meet the inclusion criteria (Figure 2). A total of 380 participants were enrolled in the study and randomized; however, 114 (29.2%) participants were lost to follow-up and were not included in the study, 3 (0.8%) participants discontinued, 6 (1.6%) participants had missing data, and 16 (4.2%) participants reported being HIV positive, leaving 241 (control group: n=122, 64.2%; intervention group: n=119, 62.6%) participants for the mITT analysis. Furthermore, 24 (20.2%) participants in the intervention group did not play the game, leaving 217 (57.1%) participants for the PP analysis (control group: n=122, 100%; intervention group: n=95, 79.8%; Figure 2).

As shown in Table 2, the characteristics of participants in the control and intervention groups were generally similar. The median age of the participants was 22 (IQR 20-24) years in both groups, and 54.9% (67/122) in the control group and 55.5% (66/119) in the intervention group were men. More than 90% (226/241) of the participants in both groups were never married, and 66.4% (81/122) in the control arm and 68.9% (82/119) in the intervention arm were currently in tertiary education. The most common recruitment platform was Facebook, accounting for 43.6% (105/241) of participants, followed by recruitment from the most popular mall in the capital city (40/241, 16.6% of participants). Participants who played the game were asked if they would recommend SwaziYolo to their peers; 98.9% (94/95) either agreed (32/95, 33.7%) or strongly agreed (62/95, 65.3%). Compared to participants who played the game, those who did not play the game were more likely to be female (14/24, 58.3% vs 10/24, 41.1%), recruited from Facebook (15/24, 62.5% vs 35/95, 36.9%), and less likely to have a tertiary education (12/24, 50% vs 70/95, 73.7%; Multimedia Appendix 2).

**Table 2:** Baseline demographics of the control and intervention groups.

Demographics		Control group (n=122)	Intervention group (n=119)
**Sex, n (%)**			
	Female	55 (45.1)	53 (44.5)
	Male	67 (54.9)	66 (55.5)
Age (y), median (IQR)		22 (20-24)	22 (20-24)
**Marital status, n (%)**			
	Single (never married and not living with a partner)	113 (92.6)	113 (95)
	Married	2 (1.6)	0 (0)
	Living with a partner	7 (5.7)	5 (4.2)
	Separated (currently not living together but not divorced)	0 (0)	1 (0.6)
**Level of education, n (%)**			
	None	2 (1.6)	3 (2.5)
	Secondary level	3 (2.5)	3 (2.5)
	High school level	36 (29.5)	31 (26.1)
	Tertiary level	81 (66.4)	82 (68.9)
**Employment status, n (%)**			
	Employed	20 (16.4)	19 (16)
	Not employed	27 (22.1)	29 (24.4)
	Student	74 (60.7)	63 (52.9)
	Self-employed	1 (0.1)	8 (6.7)
**Level of monthly income, n (%) (SZL 1=US $0.0718)**			
	<SZL 249	36 (29.5)	43 (36.1)
	SZL 250-1749	74 (60.7)	63 (52.9)
	SZL 1750-3000	12 (9.8)	13 (10.9)
	≥SZL 3000	0 (0)	0 (0)
**How did you hear about the game, n (%)**			
	Facebook	55 (45.1)	50 (42)
	Limkokwing	13 (10.7)	16 (13.4)
	Plaza Mbabane	21 (17.2)	19 (16)
	Swaziland Christian University	14 (11.5)	14 (11.8)
	Other	19 (15.6)	20 (16.8)

### Main Outcome: Risk Perception

**Figure 3:**
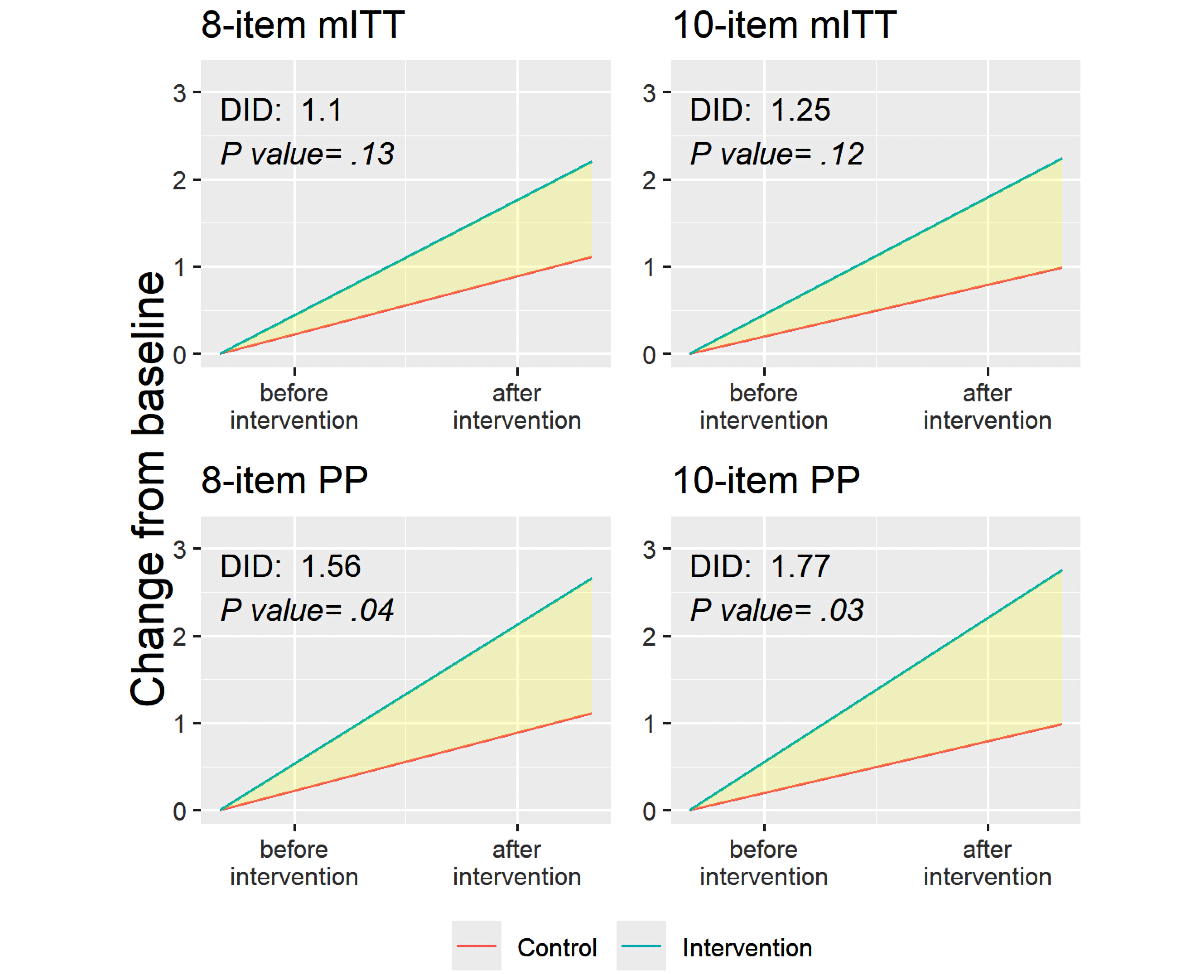
Difference-in-difference (DID) analyses of risk perception using the 8-item and 10-item indices for both modified intention-to-treat analysis (mITT) and per-protocol (PP) analyses.

In the mITT analysis, for both the 8-item and the 10-item PRHS, we observed no significant difference between the control and intervention groups in the change of the mean score for the 8-item PRHS (DID: mean 1.1, SD 0.74; *P*=.13) and the 10-item PRHS (DID: mean 1.3, SD 0.80; *P*=.12; Figure 3; Multimedia Appendix 3). However, PP analyses suggested an increase in risk perception among those who played the game in the intervention group in both PRHS indices (8-item DID mean 1.6, SD 0.74; *P*=.04 and 10-item DID mean 1.8, SD 0.83; *P*=.03; Figure 3; Multimedia Appendix 3).

Table 3 reports the results of the analyses of the secondary outcomes of protective behaviors. The odds ratios for all secondary outcomes were not statistically significant.

**Figure 4:**
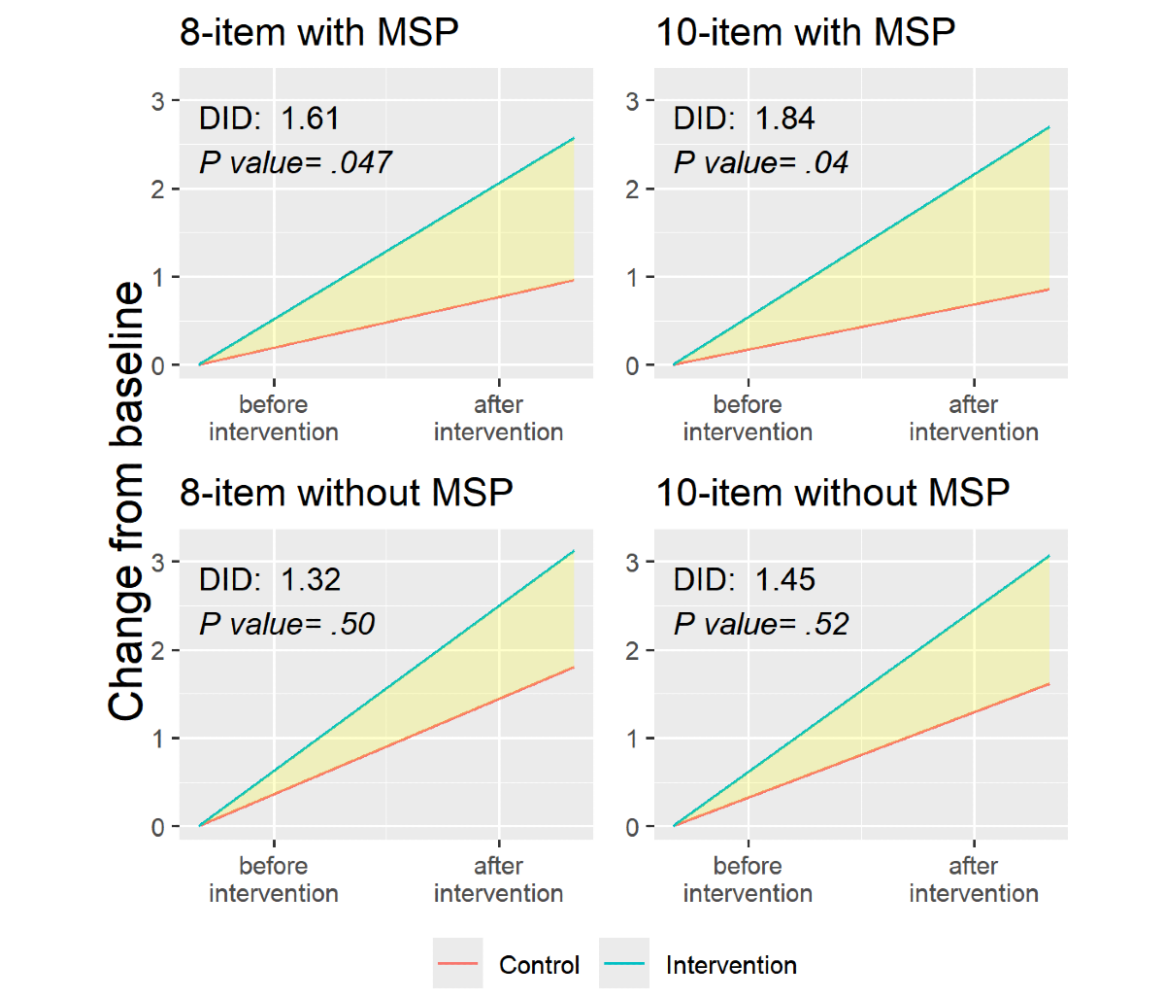
Difference-in-difference (DID) analyses of risk perception among participants with and without multiple sexual partnerships using the 8-item and 10-item indices, per-protocol analysis. MSP: multiple sexual partnerships.

The DID mean change in HIV risk perception in the 10-item PRHS was 1.8 (SD 0.89; *P*=.04) among those with multiple sexual partnerships compared to 1.4 (SD 2.22; *P*=.52) among those without multiple sexual partnerships (Figure 4; Multimedia Appendix 4). For both the 8-item and 10-item PRHS, the DID mean change scores were the highest among participants with multiple sexual partnerships.

**Figure 5:**
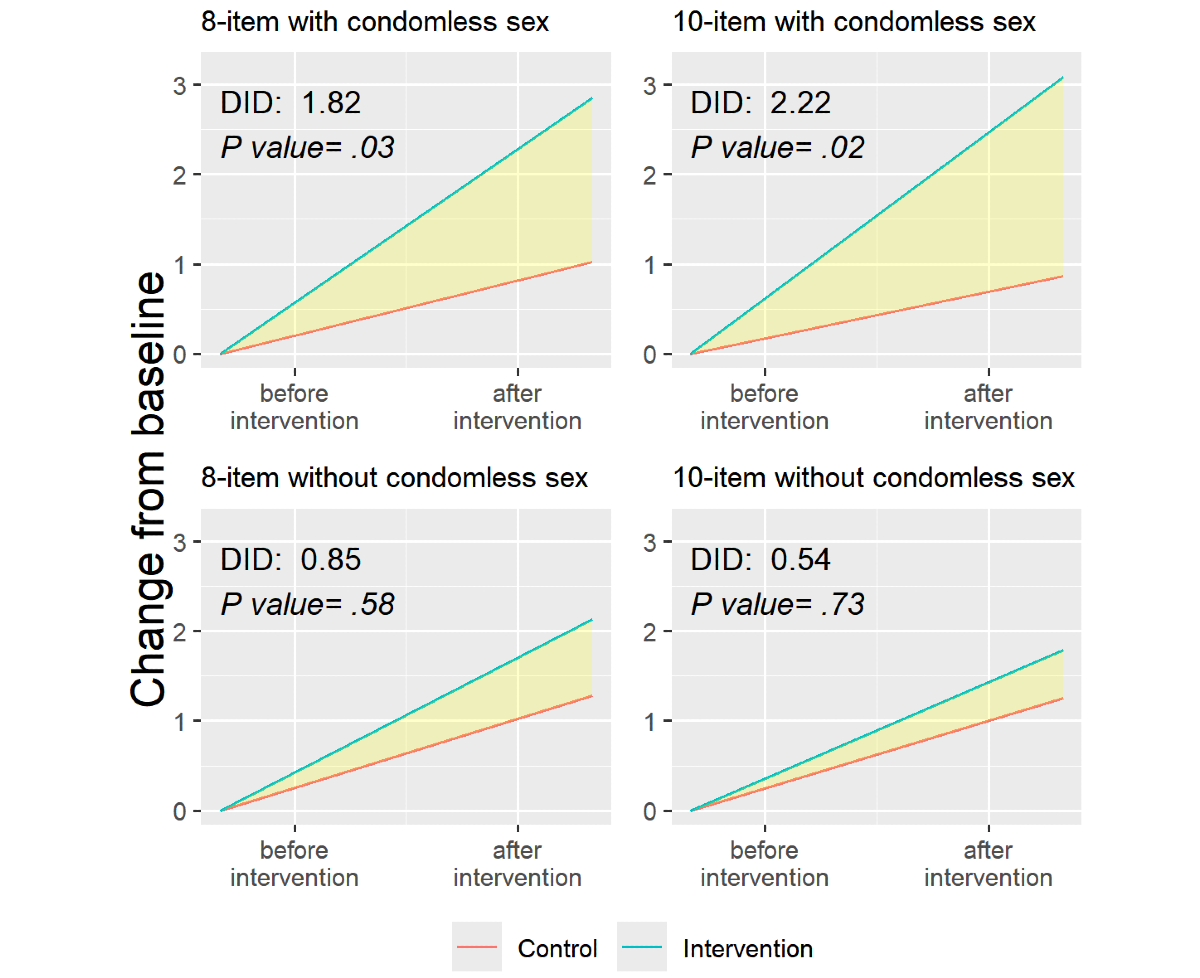
Difference-in-difference (DID) analyses of risk perception among participants with and without condomless sex using the 8-item and 10-item indices, per-protocol analysis.

Among participants reporting condomless sex, the DID mean change in HIV risk perception in the 10-item PRHS was greater (DID: mean of 2.2, SD 0.98; *P*=.02) compared to participants who did not report condomless sex (DID: mean 0.54, SD 1.59; *P*=.73; Figure 5; Multimedia Appendix 5). For both the 8-item and 10-item PRHS, the DID mean change scores were the highest among participants with condomless sex.

**Table 3:** Impact of the intervention on secondary outcomes of protective behavior.

Per-protocol secondary outcomes	Control group, n=122		Intervention group, n=95		Odds ratio (95% CI)
	Before intervention, n (%)	After intervention, n (%)	Before intervention, n (%)	After intervention, n (%)	
Number of sexual partners in the last 30 days	—^a^	23 (18.9)	—	19 (20)	1.08 (0.55-2.12)
Sex without a condom in the last 30 days	40 (32.8)	37 (30.3)	24 (25.3)	28 (29.5)	1.03 (0.57-1.88)
Condom use during the last sexual encounter	96 (78.7)	92 (75.4)	78 (82.1)	72 (75.8)	0.91 (0.45-1.85)
Tested for HIV in the last 30 days	22 (18)	19 (15.6)	13 (13.7)	17 (17.9)	1.35 (0.63-2.88)
Intention to test for HIV	10 (8.2)	17 (13.9)	9 (9.5)	14 (14.7)	1.52 (0.09-25.41)
Intention to know the HIV status of partners	109 (89.3)	107 (87.7)	78 (82.1)	83 (87.4)	1.34 (0.54-3.33)
Intention to reduce multiple concurrent partnerships	89 (73)	85 (69.7)	68 (71.6)	72 (75.8)	1.51 (0.77-2.97)
Intention to use a condom in the next sexual encounter	104 (85.2)	97 (79.5)	78 (82.1)	81 (85.3)	1.89 (0.83-4.3)
Steady partner has tested	99 (81.1)	101 (82.8)	77 (81.1)	79 (83.2)	1.04 (0.47-2.26)
Knows partner’s HIV status	88 (71.1)	86 (70.5)	64 (67.4)	65 (68.4)	1 (0.52- 1.9)

^a^Not available.

## Discussion

### HIV Risk Perception

In primary analyses of this RCT of SwaziYolo—a smartphone-based, interactive, educational story game to address HIV risk perception—the intervention was not associated with a statistically significant change in HIV risk perception among young people in Eswatini. However, the results of the PP analyses suggest that playing the game at least once significantly increased HIV risk perception.

Several factors may explain these results, with implications for the development of the next iteration of SwaziYolo and other serious game interventions. The greater effect observed in the PP analysis and the fact that 20% (24/119) of individuals randomized to the intervention group did not play the game indicate that strategies to increase the uptake of serious game interventions may increase effectiveness. The intervention duration was 30 days, and it is possible that a longer period would have increased efficacy. Challenges associated with uptake may have included carrier network failure, lack of adequate free storage on the phone, and phone resources, as reported by most participants on our online support platform. Furthermore, individuals who did not play the game were more likely to be female, recruited from Facebook, and less likely to have tertiary education. This suggests that different strategies may be needed to engage these populations, such as different game design considerations for young men and women.

### HIV Risk Perception and Sexual Behaviors

We found that the impact of the intervention was highest among subgroups reporting multiple sexual partnerships and condomless sex. This finding suggests that serious games, such as SwaziYolo, should be prioritized for people who may be more likely to benefit from interventions focused on HIV risk perceptions. Increasing HIV risk perception can lead to behavior change and increase uptake of HIV prevention interventions [20]. For example, previous studies have found that risk perception is associated with condom use [19,26], PrEP use [15,19,27,28], and HIV testing [26,29,30]. However, the effect size observed in the changes in PP HIV risk perception was modest and may not have been powerful enough to elicit intention to engage in protective behavior. Although the PRHS [20] has been validated to capture multiple dimensions of HIV risk perception, the threshold sufficient to elicit the intention to engage in behavioral change is still subject to further research in our population.

Our study was among the first to use serious games to improve HIV risk perception in a country where 24.8% of the population aged 15 years and older live with HIV [2]. Future studies should examine whether other serious game interventions improve HIV risk perception and whether improved HIV risk perception through serious game interventions is effective in increasing condom use, PrEP uptake, HIV testing, and retention in HIV care among young people in countries such as Eswatini. Studies conducted in similar settings showed that young people were likely to have inaccurate HIV risk perceptions, such that even those at high risk of HIV acquisition had lower HIV risk perceptions [31-33]. Furthermore, the complex intersection of HIV risk perception and HIV stigma remains understudied in high-epidemic settings, such as Eswatini [34,35].

### Intention to Change Behavior

Our secondary analysis assessing the impact of SwaziYolo on intention to engage in protective sexual behaviors did not show any statistical difference between groups. This may have been a result of the limited time of exposure to the interventions (4 weeks). Future interventions should assess such effects over longer durations, as the duration of exposure has been highlighted as one of the key factors for sustaining serious game intervention effects [36,37]. For example, in a 24-week follow-up of an HIV medication adherence serious game intervention, the intervention group was 3.75 times more likely to have optimal PrEP dosing compared to the control group that did not receive the intervention [38]. Another plausible reason for the limited impact of SwaziYolo on secondary behavior outcomes could be that HIV risk perception has a limited impact on behavior. However, a substantial body of research has shown that HIV risk perception can be a significant motivator for adopting protective behaviors, especially when combined with other behavioral theory–based constructs, such as self-efficacy, stigma mitigation, and access to HIV prevention tools such as condoms and PrEP [19,27,28,34,39]. It is also possible that, due to broken randomization, participants in the PP analyses had less need to significantly change their behavior. Future research is needed to examine the impact of interventions to improve HIV risk perception on behavioral change and the uptake of protective interventions.

### Player Engagement and Acceptability

At least 80% (95/119; Multimedia Appendix 2) of the participants played the game. This engagement rate is higher than that in most serious games [16] and shows the engagement of young people and the potential for future interventions to use serious games. Up to 98% (94/95) of those who played the game said they would recommend it to their peers, indicating that SwaziYolo achieved acceptance among young people in Eswatini. With increased smartphone access, processing power, and advances in artificial intelligence, we hypothesize that future serious games will achieve greater engagement and retention. For example, in 2017, smartphone ownership was 55% in South Africa (a neighboring country to Eswatini sharing many socioeconomic similarities) [34], and in 2024, smartphone ownership increased to 71% [35]. The types of smartphones available in 2024 have considerably more computing power and storage, which will support engagement, immersion, and game aesthetics.

### Limitations

First, the engagement data were based on self-reports. The game data used a proprietary architecture, which limited our access to game-generated data and did not allow us to analyze intensity (ie, exposure to different intervention components within the serious game) as generally practiced in the field [40,41]. Future studies should seek full access to data repositories for serious games to allow game paradata analytics. Second, although the characteristics of participants in the mITT analyses were generally balanced, 29.6% (56/189) of the participants in the control group and 30.8% (58/188) of the participants in the intervention group were lost to follow-up. Additionally, 4.2% (8/189) of the participants in the control group and 4.3% (8/188) of the participants in the intervention group reported being HIV positive. However, this was not identified until after the study, and these individuals were not included in the analyses, deviating from standard ITT principles. Consequently, the benefits of randomization in minimizing selection bias might have been diminished in both mITT and PP analyses. Finally, participants in the control (waitlist) group may have experienced the Hawthorne effect, becoming more aware of their behavior and modifying their HIV risk perception due to anticipation of gaining access to SwaziYolo after 4 weeks. This may have increased their risk perception scores and diminished the observed difference between the groups.

### Strengths

First, compared to other serious game interventions in sub-Saharan Africa, there was no physical contact with study participants during the intervention period apart from initial recruitment, demonstrating the potential for a fully online serious game intervention. This may have yielded a more realistic estimate of the potential adoption and proximal feasibility of technology-based HIV interventions in low- and middle-income countries. Second, overall, participants found the game highly acceptable, showing potential for future iterations of SwaziYolo and other serious game interventions to promote the uptake of HIV prevention interventions among young people in Eswatini and similar settings.

### Conclusions

In a country with high HIV prevalence, SwaziYolo was acceptable and increased HIV risk perception among young people who self-selected to play the game, especially among those who reported multiple sexual partnerships or condomless sex and who were most likely to benefit from interventions to prevent HIV. Although the mITT analysis showed no impact, the findings support the need for future research to optimize and evaluate the intervention.

## Appendix

Multimedia Appendix 1 Serious Games HIV Prevention Trial before and after intervention questionnaire.

Multimedia Appendix 2 Characteristics of participants who did and did not play the game.

Multimedia Appendix 3 Difference-in-difference analysis of risk perception.

Multimedia Appendix 4 Difference-in-difference analysis of risk perception among participants with and without multiple sexual partnerships.

Multimedia Appendix 5 Risk perception among those with and without condomless sex in the past 30 days.

Multimedia Appendix 6 CONSORT-eHEALTH checklist (version 1.6.1).

## Abbreviations

CONSORT-EHEALTH: Consolidated Standards for Reporting Trials of Electronic and Mobile Health Applications and Online Telehealth
DID: difference-in-difference
mITT: modified intention-to-treat
OTP: one-time passcode
PP: per-protocol
PrEP: pre-exposure prophylaxis
PRHS: Perceived Risk of HIV Infection Scale
RCT: randomized controlled trial
SGPrev-Trial: Eswatini (formerly Swaziland) Serious Games HIV Prevention Trial

## Data Availability

The datasets generated or analyzed during this study are not publicly available due to privacy and ethical restrictions but are available from the corresponding author on reasonable request.

## References

[1] (2022). In danger: UNAIDS global AIDS update 2022. Joint United Nations Programme on HIV/ AIDS.

[2] (2021). Eswatini population-based HIV impact assessment 3 2021 (SHIMS3 2021): final report November 2023. Ministry of Health, Eswatini.

[3] The extended national multisectoral HIV and AIDS framework (eNSF) 2014-2018. National Emergence Response Council on HIV and AIDS.

[4] Bicego GT, Nkambule R, Peterson I, Reed J, Donnell D, Ginindza H, Duong YT, Patel H, Bock N, Philip N, Mao C, Justman J (2013). Recent patterns in population-based HIV prevalence in Swaziland. PLoS One.

[5] Nkambule R, Philip NM, Reid G, Mnisi Z, Nuwagaba-Biribonwoha H, Ao TT, Ginindza C, Duong YT, Patel H, Saito S, Solmo C, Brown K, Moore CS, Voetsch AC, Bicego G, Bock N, Mhlanga F, Dlamini T, Mabuza K, Zwane A, Sahabo R, Dobbs T, Parekh BS, El-Sadr W, Ryan C, Justman J (2021). HIV incidence, viremia, and the national response in Eswatini: two sequential population-based surveys. PLoS One.

[6] Lukhele BW, Musumari P, El-Saaidi C, Techasrivichien T, Suguimoto SP, Ono Kihara M, Kihara M (2016). Efficacy of mobile serious games in increasing HIV risk perception in Swaziland: a randomized control trial (SGprev Trial) research protocol. JMIR Res Protoc.

[7] Krath J, Schürmann L, von Korflesch HF (2021). Revealing the theoretical basis of gamification: a systematic review and analysis of theory in research on gamification, serious games and game-based learning. Comput Hum Behav.

[8] Djaouti D, Alvarez J, Jessel JP, Rampnoux O, Ma M, Oikonomou A, Jain LC (2011). Origins of serious games. Serious Games and Edutainment Applications.

[9] Fiellin LE, Kyriakides TC, Hieftje KD, Pendergrass TM, Duncan LR, Dziura JD, Sawyer BG, Fiellin DA (2016). The design and implementation of a randomized controlled trial of a risk reduction and human immunodeficiency virus prevention videogame intervention in minority adolescents: PlayForward: Elm City Stories. Clin Trials.

[10] Winskell K, Sabben G, Ondeng'e K, Odero I, Akelo V, Mudhune V (2019). A smartphone game to prevent HIV among young Kenyans: household dynamics of gameplay in a feasibility study. Health Educ J.

[11] Winskell K, Sabben G, Akelo V, Ondeng'e K, Odero I, Mudhune V (2020). A smartphone game to prevent HIV among young Kenyans: local perceptions of mechanisms of effect. Health Educ Res.

[12] Haruna H, Hu X, Chu SK, Mellecker RR, Gabriel G, Ndekao PS (2018). Improving sexual health education programs for adolescent students through game-based learning and gamification. Int J Environ Res Public Health.

[13] Datta S, Burns J, Maughan-Brown B, Darling M, Eyal K (2015). Risking it all for love? Resetting beliefs about HIV risk among low-income South African teens. J Econ Behav Organ.

[14] Corneli AL, McKenna K, Headley J, Ahmed K, Odhiambo J, Skhosana J, Wang M, Agot K, FEM-PrEP Study Group (2014). A descriptive analysis of perceptions of HIV risk and worry about acquiring HIV among FEM-PrEP participants who seroconverted in Bondo, Kenya, and Pretoria, South Africa. J Int AIDS Soc.

[15] Price JT, Rosenberg NE, Vansia D, Phanga T, Bhushan NL, Maseko B, Brar SK, Hosseinipour MC, Tang JH, Bekker LG, Pettifor A (2018). Predictors of HIV, HIV risk perception, and HIV worry among adolescent girls and young women in Lilongwe, Malawi. J Acquir Immune Defic Syndr.

[16] Prata N, Morris L, Mazive E, Vahidnia F, Stehr M (2006). Relationship between HIV risk perception and condom use: evidence from a population-based survey in Mozambique. Int Fam Plan Perspect.

[17] Schaefer R, Thomas R, Maswera R, Kadzura N, Nyamukapa C, Gregson S (2020). Relationships between changes in HIV risk perception and condom use in East Zimbabwe 2003-2013: population-based longitudinal analyses. BMC Public Health.

[18] Cao Z, Chen J, Lin B, Zhang C, Zhong X (2023). Factors influencing intention on condom use during sexual intercourse with regular female partners among men who have sex with men in Western China: a structural equation modeling analysis. Sex Transm Dis.

[19] Warren EA, Paterson P, Schulz WS, Lees S, Eakle R, Stadler J, Larson HJ (2018). Risk perception and the influence on uptake and use of biomedical prevention interventions for HIV in sub-Saharan Africa: a systematic literature review. PLoS One.

[20] Napper LE, Fisher DG, Reynolds GL (2012). Development of the perceived risk of HIV scale. AIDS Behav.

[21] Lukhele BW, Techasrivichien T, Musumari PM, El-Saaidi C, Suguimoto SP, Ono-Kihara M, Kihara M (2016). Multiple sexual partnerships and their correlates among Facebook users in Swaziland: an online cross-sectional study. Afr J AIDS Res.

[22] Chu SK, Kwan AC, Reynolds R, Mellecker RR, Tam F, Lee G, Hong A, Leung CY (2015). Promoting sex education among teenagers through an interactive game: reasons for success and implications. Games Health J.

[23] Arnab S, Dunwell I, Debattista K (2013). Serious Games for Healthcare: Applications and Implications.

[24] Lukhele BW, Techasrivichien T, Suguimoto SP, Musumari PM, El-Saaidi C, Haumba S, Tagutanazvo OB, Ono-Kihara M, Kihara M (2016). Structural and behavioral correlates of HIV infection among pregnant women in a country with a highly generalized HIV epidemic: a cross-sectional study with a probability sample of antenatal care facilities in Swaziland. PLoS One.

[25] Hopewell S, Chan AW, Collins GS, Hróbjartsson A, Moher D, Schulz KF, Tunn R, Aggarwal R, Berkwits M, Berlin JA, Bhandari N, Butcher NJ, Campbell MK, Chidebe RC, Elbourne D, Farmer A, Fergusson DA, Golub RM, Goodman SN, Hoffmann TC, Ioannidis JP, Kahan BC, Knowles RL, Lamb SE, Lewis S, Loder E, Offringa M, Ravaud P, Richards DP, Rockhold FW, Schriger DL, Siegfried NL, Staniszewska S, Taylor RS, Thabane L, Torgerson D, Vohra S, White IR, Boutron I (2025). CONSORT 2025 statement: updated guideline for reporting randomized trials. Nat Med.

[26] Ngure K, Thuo N, Ogello V, Kiptinness C, Kamolloh K, Burns BF, Mugo NR, Bukusi EA, Garrison L, Baeten JM, Haberer JE (2021). Dynamic perceived HIV risk and sexual behaviors among young women enrolled in a PrEP trial in Kenya: a qualitative study. Front Reprod Health.

[27] Sewell WC, Patel RR, Blankenship S, Marcus JL, Krakower DS, Chan PA, Parker K (2020). Associations among HIV risk perception, sexual health efficacy, and intent to use PrEP among women: an application of the risk perception attitude framework. AIDS Educ Prev.

[28] Hill LM, Maseko B, Chagomerana M, Hosseinipour MC, Bekker LG, Pettifor A, Rosenberg NE (2020). HIV risk, risk perception, and PrEP interest among adolescent girls and young women in Lilongwe, Malawi: operationalizing the PrEP cascade. J Int AIDS Soc.

[29] Lin Y, Li C, Wang L, Jiao K, Ma W (2021). The mediated effect of HIV risk perception in the relationship between peer education and HIV testing uptake among three key populations in China. AIDS Res Ther.

[30] Ajayi AI, Mudefi E, Adeniyi OV, Goon DT (2019). Achieving the first of the Joint United Nations Programme on HIV/AIDS (UNAIDS) 90-90-90 targets: understanding the influence of HIV risk perceptions, knowing one's partner's status and discussion of HIV/sexually transmitted infections with a sexual partner on uptake of HIV testing. Int Health.

[31] Jung MS, Dlamini NS, Cui X, Cha K (2022). Prevalence of HIV testing and associated factors among young adolescents in Eswatini: a secondary data analysis. BMC Pediatr.

[32] Onyechi KC, Eseadi C, Okere AU, Otu MS (2016). Effects of Rational-Emotive Health Education Program on HIV risk perceptions among in-school adolescents in Nigeria. Medicine (Baltimore).

[33] Tsegay G, Edris M, Meseret S (2013). Assessment of voluntary counseling and testing service utilization and associated factors among Debre Markos University Students, North West Ethiopia: a cross-sectional survey in 2011. BMC Public Health.

[34] Machemedze T (2023). Does self-perceived HIV risk mediate the potential association between HIV-related symbolic stigma and sexual behaviour among young adult women in Cape Town, South Africa?. BMC Public Health.

[35] Embleton L, Logie CH, Ngure K, Nelson L, Kimbo L, Ayuku D, Turan JM, Braitstein P (2023). Intersectional stigma and implementation of HIV prevention and treatment services for adolescents living with and at risk for HIV: opportunities for improvement in the HIV continuum in sub-Saharan Africa. AIDS Behav.

[36] Jitmun W, Palee P, Choosri N, Surapunt T (2023). The success of serious games and gamified systems in HIV prevention and care: scoping review. JMIR Serious Games.

[37] Smith AU, Khawly GM, Jann J, Zetina AP, Padilla J, Schnall R (2023). A review of serious gaming as an intervention for HIV prevention. Curr HIV/AIDS Rep.

[38] Whiteley LB, Olsen EM, Haubrick KK, Odoom E, Tarantino N, Brown LK (2021). A review of interventions to enhance HIV medication adherence. Curr HIV/AIDS Rep.

[39] Tully S, Cojocaru M, Bauch CT (2015). Sexual behavior, risk perception, and HIV transmission can respond to HIV antiviral drugs and vaccines through multiple pathways. Sci Rep.

[40] Tan JW, Ng KB, Mogali SR (2022). An exploratory digital board game approach to the review and reinforcement of complex medical subjects like anatomical education: cross-sectional and mixed methods study. JMIR Serious Games.

[41] Lu W, Griffin J, Sadler TD, Laffey J, Goggins SP (2023). Serious game analytics by design: feature generation and selection using game telemetry and game metrics: toward predictive model construction. J Learn Anal.

