# Supplementary material for: Efficacy of a Mobile Serious Game (SwaziYolo) for Increasing HIV Risk Perception: Randomized Controlled Trial
Source: JMIR Serious Games. 2025 Nov 24;13:e70333. doi: 10.2196/70333 (PMC12686855; doi:10.2196/70333)
Supplement: Multimedia Appendix 1 [file games_v13i1e70333_app1.docx]

Research Title:

Efficacy of Serious Games in Increasing HIV Risk Perception in Swaziland: A Randomized Control Trial (SGprev Trial)

| **Pre and post intervention questionnaire for the SGpriv Trial** | | |
| --- | --- | --- |
| **#** | **QUESTION** | **RESPONSE** |
| **Socio Demographic Information** | | |
|  | What is your mobile phone number (asked this question during registration into the trial) |  |
| Q1 | How old are you? |  |
| Q2 | What is your marital status? | 1. Single (Never married and not living with a partner) 2. Married 3. Living with a partner 4. Separated (currently not living together but not divorced 5. Divorced 6. Widowed |
| Q3 | What is your current highest level of education? | 1. None 2. Primary level 3. Secondary level 4. High School level 5. Tertiary level |
| Q4 | What is your current employment status? | 1. Employed 2. Not employed 3. Student 4. Self employed |
| Q5 | What is your (personal) current total monthly income from all sources?  If no income, please select @ | 1. Less than E249 2. More than E249 but less than E1749 3. More than E1749 but less than E3000 4. More than 3000 5. @ |
| Q6 | Have you ever had an HIV test | 1. Yes 2. No |

| **Sexual reproductive history**  The following section relates to questions about sexual activity to gain a better  understanding of some reproductive health issues. Once more we would like to assure you of the confidential and anonymous nature of this survey. | | |
| --- | --- | --- |
| Q7 | Did you use a condom in your last sexual activity? | 1. Yes 2. No |

| Q8 | When was the last time you had sex without a condom | | 1. Within the past 30 days 2. 2-6 months ago 3. 7-12 months ago 4. More than 1 year ago |
| --- | --- | --- | --- |
| Q9 | How many sexual partners did you have in the past 30 days? | |  |
| **Thoughts about likelihood of getting HIV**  This section relates to what you think are your chances of getting HIV infection (PRHS) | | | |
| Q10 | | What is your gut feeling about how likely you are to get infected with HIV? | 1. Extremely unlikely 2. Very unlikely 3. Somewhat likely 4. Very likely 5. Extremely likely |
| Q11 | | I worry about getting infected with HIV | 1. None of the time 2. Rarely 3. Some of the time 4. A moderate amount of time 5. A lot of the time 6. All of the time |
| Q12 | | Picturing myself getting HIV is something I find | 1. Very hard to do 2. Hard to do 3. Easy to do 4. Very easy to do |
| Q13 | | Getting HIV is something I am… | 1. Not concerned about 2. A little concerned about 3. Moderately concerned about 4. Concerned about a lot 5. Extremely concerned about |
| Q14 | | I am sure I will NOT get HIV infected | 1. Strongly disagree 2. Disagree 3. Somewhat agree 4. Somewhat disagree 5. Agree 6. Strongly agree |
| Q15 | | I feel I am unlikely to get infected with HIV | 1. Strongly disagree 2. Disagree 3. Somewhat disagree 4. Somewhat agree 5. Agree 6. Strongly agree |
| Q16 | | I feel vulnerable to HIV infection | 1. Strongly disagree 2. Disagree 3. Somewhat disagree 4. Somewhat agree 5. Agree 6. Strongly agree |
| Q17 | | There is a chance, no matter how small, I could get HIV | 1. Strongly disagree 2. Disagree 3. Somewhat disagree 4. Somewhat agree 5. Agree 6. Strongly agree |

| Q18 | | I think my chances of getting infected with HIV are | 1. Zero 2. Almost zero 3. Small 4. Moderate 5. Large 6. Very Large |
| --- | --- | --- | --- |
| Q19 | | Getting HIV is something I have | 1. Never thought about 2. Rarely thought about 3. Thought about some of the time 4. Thought about often |
| **Intention to change your sexual behavior**  This section asks about your intention to change your sexual behavior in the coming months | | | |
| Q20 | Have you ever been tested for HIV? | | 1. Yes 2. No |
| Q21 | When were you last tested for HIV? | | 1. Within the past 30 days 2. 2-6 months ago 3. 7-12 months ago 4. More than 1 year ago |
| Q22 | Do you intend to test for HIV in the coming months? | | 1. Yes 2. No 3. No, I am already HIV Positive |
| Q23 | Do you intend to know ALL your sexual partners' HIV statuses? | | 1. Yes 2. No |
| Q24 | Do you intend to reduce your number of sexual partners in the next months? | | 1. Yes 2. No |
| Q25 | Do you Intend to use a condom in the next time you have sexual intercourse | | 1. Yes 2. No 3. I don’t know |
| This section asks about your sexual partner’s history (current partners’) if more than one partners then the last partner you had sexual intercourse with | | | |
| Q26 | Has your steady partner ever tested for HIV | | 1. Yes 2. No |
| Q27 | Do you know your steady partner’s HIV status | | 1. Yes 2. No |
| SwaziYolo game (this section will be in pre and post survey) | | | |

| Q28 | How did you hear about this game | 1. Facebook 2. Limkokwing 3. Plaza Mbabane |
| --- | --- | --- |
|  |  | 4. Swaziland Christian University |
|  |  | 5. Other (Specify) |
| **Experience with the SwaziYolo game**  This next section asks about your experience with the SwaziYolo game (these questions asked at post intervention only to those who are randomized to play SwaziYolo) | | |
| Q29 | How many times did you play SwaziYolo from start to end? | 1. Once but did not get to the end 2. Once and I got to the end 3. Two times and I got to the end in both times 4. More than two times |
| Q30 | I would recommend the SwaziYolo game to my friends | 1. Strongly agree 2. Agree 3. Disagree 4. Strongly disagree |
| Q31 | On a scale 1 – 5 (one being the list and 5 being the most) how satisfied are you with this game |  |
| Q32 | How did you hear about this game | 1. Facebook 2. Limkokwing 3. Plaza Mbabane 4. Swaziland Christian University 5. Other (Specify) |
| Pre-Intervention closing message  Thank you for taking part in this survey, you may now continue to play the SwaziYolo game. Please click here to download the game and you will use this code ***** to play the game. (was seen only by those randomized to the intervention group)  Pre-Intervention closing message  Thank you for taking part in this survey, you have been selected to be in the waiting list. You will be contacted after four weeks to play the SwaziYolo game. (was seen only by those randomized to the wait-list control group) | | |
| Post intervention closing message  Thank you for taking part in this survey you will be informed when the latest update of the SwaziYolo game in available. (was seen only by those randomized to the intervention group)  Post intervention closing message  Thank you for taking part in this survey you may now continue to play the SwaziYolo game. Please click here (was seen only by those randomized to the wait-list control group) | | |
