# Supplementary material for: Efficacy of a Mobile Serious Game (SwaziYolo) for Increasing HIV Risk Perception: Randomized Controlled Trial
Source: JMIR Serious Games. 2025 Nov 24;13:e70333. doi: 10.2196/70333 (PMC12686855; doi:10.2196/70333)
Supplement: Multimedia Appendix 2 [file games_v13i1e70333_app2.docx]

|  |  | **Did not play the game (24)** | **Played game at least once (95)** |
| --- | --- | --- | --- |
|  |  | **n (%)** | **n (%)** |
| **Demographics** |  |  |  |
| **Gender** | Female | 14 (58.33) | 39 (41.05) |
|  | Male | 10 (41.67) | 56 (58.95) |
|  |  |  |  |
| **Age** | **Median (IQR)** | 22 (4.00) | 22 (4.00) |
|  |  |  |  |
| **Marital status** |  |  |  |
|  | 1. Single (Never married and not living with a partner) | 24 (100) | 89 (93.68) |
|  | 2. Married |  |  |
|  | 3. Living with a partner | 0 | 5 (5.26) |
|  | 4. Separated (currently not living together but not divorced) | 0 | 1 (1.05) |
|  |  |  |  |
| **Level of education** |  |  |  |
|  | 1. None | 1 (4.17) | 2 (2.11) |
|  | 3. Secondary level | 2 (8.33) | 1 (1.05) |
|  | 4. High School level | 9 (37.50) | 22 (23.16) |
|  | 5. Tertiary level | 12 (50.00) | 70 (73.68) |
|  |  |  |  |
| **Employment status** |  |  |  |
|  | 1. Employed | 6 (25.00) | 13 (13.68) |
|  | 2. Not employed | 5 (20.83) | 24 (25.26) |
|  | 3. Student | 12 (50.00) | 51 (53.68) |
|  | 4. Self employed | 1 (4.17) | 7 (7.37) |
|  |  |  |  |
| **Level of monthly income** |  |  |  |
|  | 1. Less than E249 | 8 (33.33) | 35 (36.84) |
|  | 2. More than E249 but less than E1749 | 15 (62.50) | 48 (50.53) |
|  | 3. More than E1749 but less than E3000 | 1 (4.17) | 12 (12.63) |
|  |  |  |  |
| **How did you hear about game** |  |  |  |
|  | Facebook | 15 (62.50) | 35 (36.84) |
|  | Limkokwing | 1 (4.17) | 15 (15.79) |
|  | Plaza Mbabane | 3 (12.50) | 16 (16.84) |
|  | SCU | 2 (8.33) | 12 (12.63) |
|  | Other | 3 (12.50) | 17 (17.89) |
|  |  |  |  |
| **I would recommend SwaziYolo to friends** |  |  |  |
|  | **Strongly disagree** | 2 (8.33) | 1 (1.05) |
|  | **Disagree** | 0 | 0 |
|  | **agree** | 12 (50.00) | 32 (33.68) |
|  | **Strongly agree** | 10 (41.67) | 62 (65.26) |
|  |  |  |  |
| **Condom use at last sex (baseline)** |  |  |  |
|  | **Yes** | 21 (87.50) | 78 (82.11) |
|  |  |  |  |
| **Risk perception (8-item, baseline)** | **Median (IQR)** | 14 (7.50) | 14 (10.00) |
| **Risk perception (10-item, baseline)** | **Median (IQR)** | 19 (9.00) | 18 (11.00) |
