# Supplementary material for: Efficacy of a Mobile Serious Game (SwaziYolo) for Increasing HIV Risk Perception: Randomized Controlled Trial
Source: JMIR Serious Games. 2025 Nov 24;13:e70333. doi: 10.2196/70333 (PMC12686855; doi:10.2196/70333)
Supplement: Multimedia Appendix 3 [file games_v13i1e70333_app3.docx]

| ***Risk perception** | Control (122) | | | | Intervention (119) | | | |  |  |
| --- | --- | --- | --- | --- | --- | --- | --- | --- | --- | --- |
|  | **Pre** | **Post** | **Mean Diff** | | **Pre** | **Post** | **Mean Diff** | | **DID** | |
| **Risk perception data ITT** | **Mean** | **Mean** |  | **P value** | **Mean** | **Mean** |  | **P value** | **Mean** | **P value** |
| 8-item index | 14.01 | 15.11 | 1.11 | 0.03 | 13.66 | 15.87 | 2.21 | <.0001 | 1.10 | 0.13 |
| 10-item index | 18.47 | 19.46 | 0.99 | 0.08 | 17.83 | 20.08 | 2.24 | <.0001 | 1.25 | 0.12 |
| **Risk perception data PP** | Control (122) | | | | Intervention (95) | | | |  |  |
| 8-item index | 14.01 | 15.11 | 1.11 | 0.03 | 13.43 | 16.09 | 2.66 | <.0001 | 1.56 | 0.04 |
| 10-item index | 18.47 | 19.46 | 0.99 | 0.07 | 17.60 | 20.36 | 2.76 | <.0001 | 1.77 | 0.03 |
| ^ITT = Intention-To-Treat Analysis^ | | | | | | | | | | |
| ^PP = Per-Protocol Analysis^  ^DID= Difference in Difference^ | | | | | | | | | | |
