# Supplementary material for: Efficacy of a Mobile Serious Game (SwaziYolo) for Increasing HIV Risk Perception: Randomized Controlled Trial
Source: JMIR Serious Games. 2025 Nov 24;13:e70333. doi: 10.2196/70333 (PMC12686855; doi:10.2196/70333)
Supplement: Multimedia Appendix 4 [file games_v13i1e70333_app4.docx]

| **Risk perception among those with MSPs** | Control (101) | | | | Intervention (80)  | | | |  | | |
| --- | --- | --- | --- | --- | --- | --- | --- | --- | --- | --- | --- |
|  | **Pre** | **Post** | **Mean Diff** | | **Pre** | **Post** | **Mean Diff** | | **DID** | | |
|  | **Mean** | **Mean** |  | **P value** | **Mean** | **Mean** |  | **P value** | **Mean** | **P value** | |
| 8-item index | 13.72 | 14.68 | 0.96 | 0.075 | 13.13 | 15.70 | 2.58 | <.0001 | 1.61 | 0.047 | |
| 10-item index | 18.14 | 19.00 | 0.86 | 0.15 | 17.18 | 19.88 | 2.70 | <.0001 | 1.84 | 0.04 | |
| **Risk perception among those without MSPs** | Control (21) | | | | Intervention (15)  | | | |  |  | |
| 8-item index | 15.38 | 17.19 | 1.81 | 0.16 | 15.07 | 18.20 | 3.13 | 0.04 | 1.32 | 0.50 | |
| 10-item index | 20.05 | 21.67 | 1.62 | 0.27 | 19.87 | 22.93 | 3.07 | 0.08 | 1.45 | 0.52 | |
| ^MSP =Multiple Sexual Partnerships^ | | | | | | | | | | | |
| ^DID= Difference in Difference^ | | | | | | | | | | |  |
|  | | | | | | | | | | |  |
