# Supplementary material for: Efficacy of a Mobile Serious Game (SwaziYolo) for Increasing HIV Risk Perception: Randomized Controlled Trial
Source: JMIR Serious Games. 2025 Nov 24;13:e70333. doi: 10.2196/70333 (PMC12686855; doi:10.2196/70333)
Supplement: Multimedia Appendix 5 [file games_v13i1e70333_app5.docx]

| **Risk perception and with condomless sex** | Control (82) | | | | Intervention (71)  | | | |  | |
| --- | --- | --- | --- | --- | --- | --- | --- | --- | --- | --- |
|  | **Pre** | **Post** | **Mean Diff** | | **Pre** | **Post** | **Mean Diff** | | **DID** | |
|  | **Mean** | **Mean** |  | **P value** | **Mean** | **Mean** |  | **P value** | **Mean** | **P value** |
| 8-item index | 13.44 | 14.46 | 1.02 | 0.08 | 13.31 | 16.15 | 2.85 | <.0001 | 1.82 | 0.03 |
| 10-item index | 17.93 | 18.79 | 0.87 | 0.20 | 17.28 | 20.37 | 3.08 | <.0001 | 2.22 | 0.02 |
| **Risk perception and without condomless sex** | Control (40) | | | | Intervention (24)  | | | |  |  |
| 8-item index | 15.18 | 16.45 | 1.28 | 0.18 | 13.79 | 15.92 | 2.13 | 0.08 | 0.85 | 0.58 |
| 10-item index | 19.58 | 20.83 | 1.25 | 0.20 | 18.54 | 20.33 | 1.79 | 0.16 | 0.54 | 0.73 |

|  |
| --- |
| ^DID= Difference in Difference^ |
